# Human Pegivirus Type 1 Prevalence in the General Population, Type 2 Diabetes and Cancer Patients in Taizhou, Jiangsu, China

**DOI:** 10.3390/pathogens15070706

**Published:** 2026-07-04

**Authors:** Xiuli Zhao, Yinling Li, Ziyan Wang, Zhenzhou Wan, Chiyu Zhang

**Affiliations:** 1 Medical Laboratory of Taizhou Fourth People’s Hospital, Taizhou 225300, China; angel033096@126.com (X.Z.); liyinling2008@163.com (Y.L.); wangziyan329@163.com (Z.W.); 2 Shanghai Public Health Clinical Center, Fudan University, Shanghai 201508, China; 3 School of Medicine, Anhui University of Science and Technology, Huainan 232001, China

**Keywords:** HPgV-1, healthy individual, type 2 diabetes, cancer, age, adult

## Abstract

This study aimed to investigate the prevalence of human pegivirus type 1 (HPgV-1) among healthy individuals, type 2 diabetes patients, and cancer patients in Taizhou, Jiangsu, China. A total of 2872 participants, including 2052 healthy individuals, 373 type 2 diabetes patients, and 447 cancer patients, were enrolled at Taizhou Fourth People’s Hospital between 2022 and 2024. Serum samples were collected from each participant, and HPgV-1 RNA was detected using a reverse transcription-polymerase chain reaction (RT-PCR) assay. The positive rates were compared across the three groups. The age range of healthy individuals was 5–91 years, while that of type 2 diabetes and cancer patients was 25–85 years and 34–91 years, respectively. The prevalence of HPgV-1 among healthy individuals was 15.3% (313/2052), which was significantly higher than that in type 2 diabetes patients (11.0%, 41/373, *p* < 0.05) and cancer patients (9.2%, 41/447, *p* < 0.001). In healthy individuals, the prevalence of HPgV-1 increased from childhood to young adulthood, peaking in the 19–40 years age group, followed by a decline after 40 years of age, indicating an age-related pattern. Similarly, HPgV-1 load increased from childhood to adulthood. No significant gender differences in HPgV-1 prevalence were observed across the three cohorts. In conclusion, HPgV-1 infection exhibits an age-dependent prevalence, with the lowest rate in children and adolescents and the highest in young adults (19–40 years). The significantly lower prevalence of HPgV-1 in type 2 diabetes and cancer patients raises the intriguing question of whether HPgV-1 infection may play a protective or contributory role in the pathogenesis of diabetes and cancers.

## 1. Introduction

Human pegivirus (HPgV) belongs to the genus *Pegivirus* of the family *Flaviviridae* and was classified into HPgV-1 and HPgV-2 [1]. HPgV is efficiently transmitted via percutaneous injuries (PIs), blood transfusion, sexual contact and vertical route. HPgV-1 was formerly known as GB virus C or hepatitis G virus [1]. Since its discovery, numerous epidemiological studies have confirmed that HPgV-1 is widely distributed worldwide [2]. HPgV-1 has a global prevalence with a high prevalence among HIV-1 and/or HCV-infected individuals (e.g., injection drug users and sexual transmission groups) due to sharing the same transmission routes with the two viruses [2,3]. HPgV-1 prevalence in healthy blood donors has been widely investigated and varies across different countries/regions [2,4,5]. However, HPgV-1 prevalence in the general population, especially among adolescents and the elderly, has rarely been investigated, and whether its prevalence is associated with age remains unclear.

HPgV-1 shares approximately 50% nucleotide sequence identity and about 30% amino acid sequence identity with HCV [1]. However, HPgV-1 has not been confirmed as a definitive pathogen for any human diseases [1,6]. In contrast, an increasing number of studies have focused on its interactions with the human immune system, particularly in the context of co-infection with HIV-1 or Ebola virus, where it exerts beneficial effects by attenuating the progression of AIDS or Ebola virus disease [7,8,9]. Although HPgV-1 itself does not appear to be directly pathogenic, its epidemiological distribution patterns across populations with different health conditions may provide insights into the understanding of the symbiotic relationship between the virus and its host [10,11]. For instance, whether the presence or absence of the virus carries specific clinical implications in the context of immunosuppression or chronic diseases remains poorly understood. In this study, we conducted a cross-sectional study to investigate HPgV-1 infection in a large cohort of healthy individuals, patients with type 2 diabetes, and various cancers, and assessed the association of HPgV-1 infection with sex, age, diabetes and cancers.

## 2. Materials and Methods

### 2.1. Study Subjects

A total of 2872 participants, including 2052 healthy individuals, 373 type 2 diabetes patients, and 447 cancer patients, were recruited at Taizhou Fourth People’s Hospital between 2022 and 2024. Blood was collected from healthy individuals when they visited the hospital for routine health examinations and from patients during their clinical visits. Healthy individuals were defined as those who had no acute diseases, no diabetes and no cancers at enrollment. The older healthy adults should also meet the Chinese Criteria for Healthy Older Adults (WS/T802-2022), which requires intact self-care ability, absence of severe organic diseases, and basically normal cognitive function. The type 2 diabetes patients were diagnosed according to World Health Organization criteria, and the cancer patients were diagnosed and confirmed by histopathology. All participants were screened for HIV antibodies, HCV antibodies, and HBsAg via magnetic particle chemiluminescence immunoassay (Autobio, Zhengzhou, China). Individuals who tested positive for any of these three blood-borne viruses were excluded from this study.

The study was approved by the Ethics Committee of Taizhou Fourth People’s Hospital (EC/TZFH-035), and all data were analyzed anonymously. Clinical data for all participants were retrieved from their electronic medical records.

### 2.2. HPgV-1 RNA Detection

Viral RNA was extracted from 200 μL of serum using the T183 nucleic acid extraction/purification kit (Xi’an Tianlong Technology Co., Xi’an, China) according to the manufacturer’s instructions. HPgV-1 RNA was detected using the One Step PrimeScript™ RT-PCR kit (TaKaRa, Dalian, China) with specific primers targeting a portion of the 5′ untranslated region (5′ UTR) of HPgV-1 (GenBank: NC_001710.1) [12]. The forward and reverse primers were 5′-GGCCAAAAGGTGGTGGATG-3′ (163–181 nt in HPgV-1 genome) and 5′-CTAGCGCGGYGCTTTATT-3′ (343–361 nt), respectively. The probe was 5′-CAGGGTTGGTAGGTCGTAAATCCCGGT-3′ with a FAM fluorophore and a BHQ1 quencher at its 5′- and 3′-ends, respectively [13]. The amplicon size is 199 bp. RT-qPCR was performed using the ABI 7500 system.

### 2.3. Data Analysis

Data were analyzed using SPSS version 26.0. Categorical variables are presented as counts and percentages (%). Differences in positive rates between groups were evaluated using the Pearson chi-square test. Comparisons among multiple groups were performed using one-way analysis of variance (ANOVA) with GraphPad Prism 5. A *p*-value of <0.05 was considered statistically significant.

## 3. Results

### 3.1. Study Cohorts

The age range of healthy individuals was 5–91 years, and those of patients with type 2 diabetes and cancers were 25–85 years and 34–91 years, respectively (Table 1). The healthy cohort had a balanced age distribution with ages of <18 (17.0%), 19–30 (19.5%), 31–40 (17.6%), 41–50 (17.9%), 51–60 (21.4%), and >60 (6.5%) years old. The vast majority of the type 2 diabetes and cancer patients were aged >50 years (diabetes: 81.5%, cancers: 84.6%), especially >40 years (diabetes: 92.8%, cancers: 99.8%). Most healthy individuals are female (60.6%), whereas most type 2 diabetes and cancer patients are male (57.4% and 57.5%, respectively). 

### 3.2. HPgV-1 Prevalence

**Figure 1:**
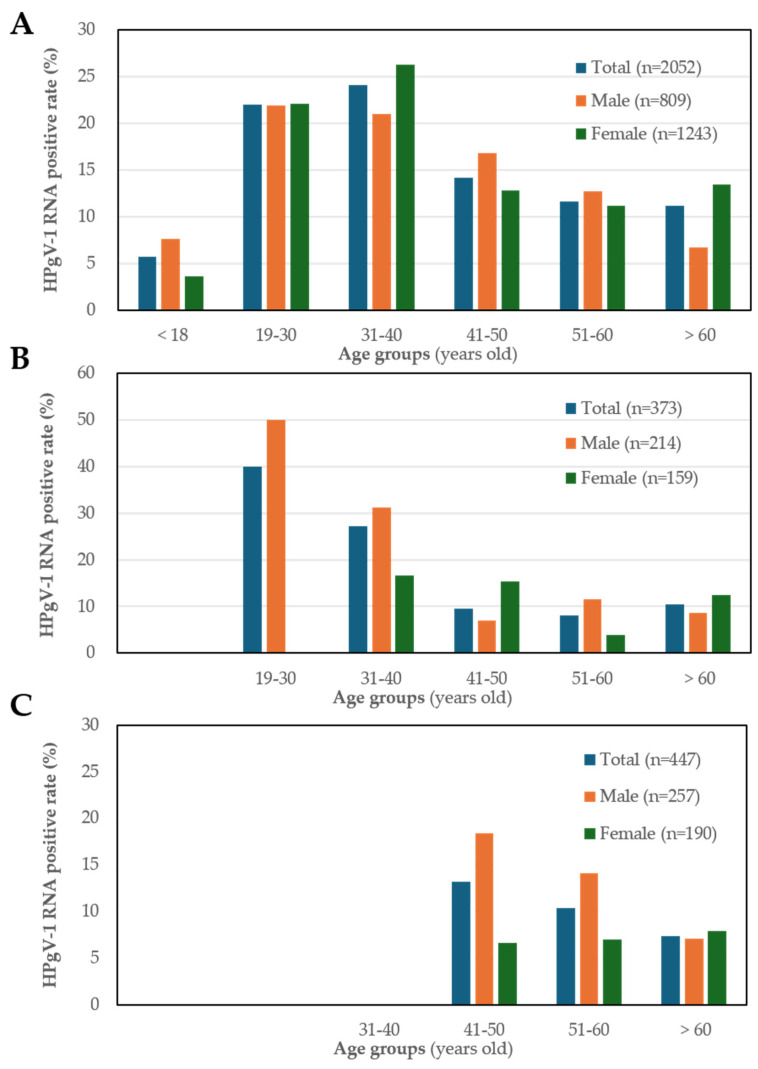
HPgV-1 prevalence in different age groups of healthy people (**A**), diabetes patients (**B**) and cancer patients (**C**). In panel (**B**), the unusually high HPgV-1 positive rate in the 19–30 years age group of diabetes patients might be due to too small sample size. In panel (**C**) (cancer patients), no analysis was performed for the age group of 31-40 years due to the inclusion of only one female patient.

The overall prevalence of HPgV-1 was 15.3% (313/2052) in healthy people across all age groups (Table 2). The highest and lowest prevalence of HPgV-1 were observed in healthy adults with ages of 19–40 years (23.0%), and children and adolescents aged 5–18 years (5.7%), respectively (Figure 1A). Interestingly, the prevalence of HPgV-1 exhibited an age-dependent pattern in healthy individuals, with a peak (24.1%) at the age group of 31–40 years (Figure 1A). There was a significant decline in HPgV-1 prevalence after the age of 40 years (*p* < 0.01), followed by a further slow decline with age.

The prevalence of HPgV-1 was 11.0% and 9.2% in type 2 diabetes and cancer patients, respectively, significantly lower than that in healthy people (Table 2). Given that the vast majority of diabetes and cancer patients were aged above 40 years, we further compared the HPgV-1 prevalence among the three groups with age over 40 years. HPgV-1 prevalence was 9.5% (*p* = 0.135) and 9.2% (*p* = 0.069) in diabetes and cancer patients older than 40 years, respectively, also substantially lower than 12.5% in the same age group of healthy people (Table 2). 

Among cancer patients, digestive cancers, respiratory cancers, urinary and male reproductive cancers, female reproductive cancers, breast cancers, endocrine cancers, and hematolymphoid cancers accounted for 50.6%, 21.9%, 8.0%, 2.2%, 8.1%, 4.9%, and 4.3%, respectively (Table 3). HPgV-1 positive rates among patients with digestive cancers, respiratory cancers, urinary and male reproductive cancers, female reproductive cancers, breast cancers, endocrine cancers, and hematolymphoid cancers were 8.9%, 11.2%, 8.3%, 10.0%, 8.3%, 13.6%, and zero, respectively (Table 3). A declining trend of HPgV-1 prevalence with age was also observed in diabetes and cancer patients, similar to the observation in healthy adults (Figure 1B,C).

No significant difference in HPgV-1 prevalence was observed between the type 2 diabetes and cancer patients, as well as between males and females across all groups (Figure 1).

### 3.3. Ct Values of HPgV-1 by the qPCR Assay

**Figure 2:**
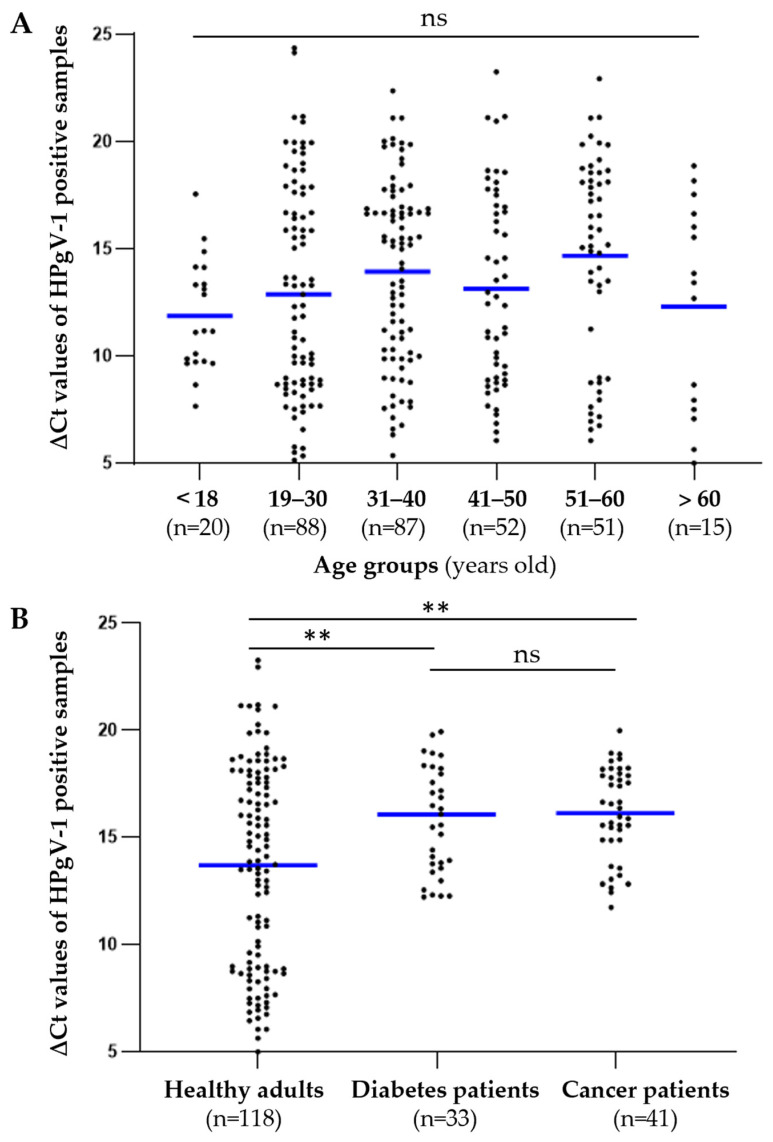
Comparisons of ∆Ct values of HPgV-1 positive samples among different age groups of healthy people (**A**), and among three age-matched cohorts (healthy people, diabetes patients and cancer patients) (**B**). The three cohorts in panel (**B**) are age-matched, with ages over 40 years. The RT-qPCR amplification of HPgV-1 was performed with 40 cycles, and the ∆Ct value is calculated as 40 minus the Ct value. The blue lines indicate the median ∆Ct values. Comparisons among groups were performed using one-way ANOVA. **, *p* < 0.01; ns, not significant.

The Ct values by qPCR were significantly negatively correlated with template input. In this study, the RT-qPCR reaction was set to run for 40 cycles, and the ∆Ct value, which is calculated as 40 minus the Ct value, is linearly positively correlated with the log HPgV-1 viral load. We compared HPgV-1 ∆Ct values among HPgV-1-positive healthy individuals across different age groups. The ∆Ct values appeared to increase from childhood to adulthood, reached the highest level in the age group of 51–60 years, and substantially declined beyond 60 years (Figure 2A). The results indicated HPgV-1 load initially increased from childhood to adulthood, followed by a decline after 60 years.

We further compared the HPgV-1 ∆Ct values among healthy individuals, type 2 diabetes and cancer patients with age over 40 years. Significantly lower ∆Ct values were observed in healthy individuals (median: 14.3) than in type 2 diabetes (median: 16.1) and cancer patients (median: 16.4) (both *p* < 0.01) (Figure 2B), implying a lower HPgV-1 load in positive healthy individuals.

## 4. Discussion

In this study, we reported 15.3%, 11.0% and 9.2% prevalence of HPgV-1 RNA in healthy people, type 2 diabetes patients and cancer patients, respectively. An age-related pattern of HPgV-1 prevalence was observed in healthy people, with an initial increase from childhood to young adulthood, and a decrease after 40 years old.

As a blood-borne virus, HPgV-1 is transmitted by percutaneous injuries (PIs), contaminated blood and/or blood products, sexual contact, and vertical mother-to-child transmission (MTCT) [2,3]. HPgV-1 was rarely detected in neonatal blood [14]. HPgV-1 infection during childhood may occur mainly via PIs and MTCT, and depend on the presence of HPgV-1 in their parents and close relatives. Relatively fewer exposure opportunities may explain the observation of the lowest HPgV-1 prevalence in healthy adolescents. The highest HPgV-1 prevalence in young adulthood (19–40 years) may be attributed to sexual transmission during their sexually active period. HPgV-1 viremia is typically cleared within several years after infection in healthy individuals. The decrease in HPgV-1 prevalence after 40 years old may be associated with the clearance of the virus by the immune system and/or decreased sexual exposure to HPgV-1 infection.

One strength of this study is that 2052 healthy individuals were enrolled. The HPgV-1 prevalence among healthy individuals in Taizhou, China, was 15.3%, markedly higher than the global average of approximately 3.1% [4]. Similarly, high HPgV-1 prevalence (>10%) was previously observed in blood donors in several Asian countries/regions (e.g., 13.7% in Qatar, 24.6% in Kuwait, and 15.8% in Hangzhou, China) [15,16,17,18]. The discrepancy may reflect the substantial geographical heterogeneity in HPgV-1 prevalence [2,15].

Previous studies and meta-analyses have reported a significant association between HPgV-1 infection and lymphoma (especially non-Hodgkin lymphoma), suggesting that HPgV-1 infection may be positively correlated with the risk of lymphoma [19,20]. Nevertheless, the association between HPgV-1 viremia and lymphoma remains controversial [2]. Distinct from previous studies, no HPgV-1 viremia was detected in patients with hematologic and lymphatic cancers in this study. The main reason may be the small sample size of hematologic and lymphatic cancer patients.

Age-matched (>40 years old) comparisons showed substantially lower HPgV-1 RNA prevalence in type 2 diabetes and cancer patients than in healthy adults. Although lower prevalence of HPgV-1 in these patients did not imply an association of HPgV-1 infection with the risk of cancers and diabetes, it at least raises the interesting question whether HPgV-1 infection plays a role in other diseases other than viral infectious diseases [10]. HPgV-1 infection modulates host immune responses with a variety of mechanisms, including reducing immune activation and inflammation, and therefore contributes to the maintenance of immune homeostasis [2,5,7]. The developments of cancer (carcinogenesis) and type 2 diabetes are attributed to completely different mechanisms, but both are associated with a dysregulated immune system and chronic inflammation [21,22]. Given the immunomodulatory activities and the beneficial clinical effects of HPgV-1 coinfection on several viral infections such as HIV-1 [2,5,7], whether persistent HPgV-1 infection also has a beneficial effect on the developments of type 2 diabetes and/or various cancers by diverse immunomodulatory mechanisms remains to be determined. On the other hand, whether the lower HPgV-1 prevalence in type 2 diabetes and cancer patients is ascribed to the suppression or inhibition of viral replication by certain medications and chemotherapy also deserves investigation.

There are limitations in this study. First, this was a single-center cross-sectional study. Given geographical heterogeneity in HPgV-1 prevalence, our findings may not be generalized to other populations or regions. The cross-sectional study design limits us from determining the dynamics of HPgV-1 infection and its association with the development of diabetes or malignant tumors. Second, the lack of detailed clinical information of these patients also limits us from exploring the potential role of HPgV-1 in disease progression. Furthermore, the lack of HPgV-1 genotyping data presents another limitation of this study. Therefore, future multicenter prospective longitudinal studies are required to determine the association and role of HPgV-1 infection in diabetes and various cancers.

## Figures and Tables

**Table 1:** Cohorts and HPgV-1 prevalence.

Item	Healthy People (%)	Diabetes Patients (%)	Cancer Patients (%)
Gender			
Male	809 (39.4)	214 (57.4)	257 (57.5)
Female	1243 (60.6)	159 (42.6)	190 (42.5)
Age (years old)			
5–15	349 (17.0)	0 (0)	0 (0)
19–30	400 (19.5)	5 (1.3)	0 (0)
31–40	361 (17.6)	22 (5.9)	1 (0.2)
41–50	368 (17.9)	42 (11.3)	68 (15.2)
51–60	440 (21.4)	112 (30.0)	135 (30.2)
≥61	134 (6.5)	192 (51.5)	243 (54.4)
HPgV-1 positive *	313 (15.3)	41 (11.0)	41 (9.2)
Total	2052	373	447

* The positive rates are shown in parentheses.

**Table 2:** HPgV-1 RNA positive rate among three cohorts.

Cohorts	Age Groups	Total	Positive	Positive Rate (%)	*p* Value
Healthy people	Overall	2052	313	15.3	NA
	>40 years old	942	118	12.5	NA
T2D patients	Overall	373	41	11.0	<0.05
	>40 years old	346	33	9.5	0.1353
Cancer patients	Overall	447	41	9.2	<0.001
	>40 years old	446	41	9.2	0.0686

T2D, type 2 diabetes; NA, not applicable.

**Table 3:** HPgV-1 positive rate among patients with different types of cancer.

Cancers	Number (%)	Positive Case	Positive Rate (%)
Digestive cancers	226 (50.6)	20	8.9
Respiratory cancers	98 (21.9)	11	11.2
Urinary and male reproductive cancers	36 (8.0)	3	8.3
Female reproductive cancers	10 (2.2)	1	10.0
Breast cancers	36 (8.1)	3	8.3
Endocrine cancers	22 (4.9)	3	13.6
Hematologic and lymphatic cancers	19 (4.3)	0	0

## Data Availability

The original contributions presented in this study are included in the article. Further inquiries can be directed to the corresponding author(s).
